# A Dual-Band Filter Using a Multimode Resonator with Asymmetrically Loaded Open-Circuited Stubs for Independent Passband Control

**DOI:** 10.3390/mi17030281

**Published:** 2026-02-25

**Authors:** Qun Chen, Li Zhang, Liqin Liu

**Affiliations:** College of Artificial Intelligence, Putian University, Putian 351100, China

**Keywords:** multimode resonator, dual-band, filter, low insertion loss

## Abstract

A novel multimode resonator is designed in this paper. Incorporating two open-circuited stubs of distinct impedances loaded onto a uniform half-wavelength transmission line, the resonator enables the realization of a dual-band filter—with center frequencies at 2.6 GHz and 4.8 GHz—through both simulation and experimental measurement. Regarding the first passband centered at 2.6 GHz, the device exhibits a return loss |S_11_| of 13.7 dB, an insertion loss |S_21_| of 0.37 dB, and a 3 dB bandwidth of 17.3%. As for the second passband with a center frequency of 4.8 GHz, the measured return loss |S_11_| amounts to 23.6 dB, the insertion loss |S_21_| measures 0.77 dB, and the 3 dB bandwidth is recorded at 8.75%. Specifically designed for 5G communication systems, the filter achieves three transmission zeros by adopting electrical coupling and 0° feeding, resulting in high selectivity and high isolation. Practical measurements verify that the experimental results are consistent with the simulation results.

## 1. Introduction

Amid the fast-paced evolution of information and communication technologies, wireless communication systems have achieved notable advancements. As a crucial component in mobile communication systems, filters are primarily responsible for frequency selection during signal transmission and reception at the transmitter and receiver, thereby filtering out interference and clutter signals. At the same time, spectral resources are growing progressively scarce. To enhance the utilization efficiency of such resources, the design of multi-band filters—including dual-band filters [1,2,3], tri-band filters, and quad-band filters [4,5,6,7]—has become increasingly crucial.

Conventional multi-band filters are constructed by cascading several single-band filters, which necessitates matching components such as duplexers. This not only adds to the system’s complexity but also increases its physical dimensions. In contrast, the use of dual-band filters can reduce system volume and minimize losses. In recent years, various methods have been proposed for designing dual-band filters. In [8], a balanced dual-band filter is designed using resonators composed of two identical folded third-order stepped impedance resonators (TSIRs). In [9], an independently reconfigurable dual-band filter based on stub-loaded multimode resonators is proposed. By analyzing the operating modes of the stepped impedance resonator (SIR), the optimal positions of the loaded stubs and varactors are determined, and a 0° feeding structure is introduced, ultimately achieving bandpass filtering functionality with independently reconfigurable center frequencies of the dual passbands. In [10], a novel microstrip switchable bandpass filter (BPF) is presented. The topology realizes three filtering mode switches (broadband bandpass filter, dual-band bandpass filter, and tri-band bandpass filter) by controlling the on-off state of radio frequency PIN diodes. In [11], a dual-band microstrip bandpass filter with a unique structure is designed, adopting broadside coupling technology and a feeding method based on a 50 Ω coplanar waveguide, achieving a compact structure, narrow fractional bandwidth, and low insertion loss. In [12], a self-coupled resonant cavity composed of asymmetric parallel microstrip transmission lines is proposed, and a dual-band bandpass filter with a wide stopband is designed and implemented. In [13], a design of a center-loaded stub stepped impedance resonator based on a T-type resonator is proposed, and a fourth-order dual-band bandpass filter is realized on a double-sided YBCO high-temperature superconducting thin film with excellent performance. While it achieves low IL (0.41/0.65 dB after scaling), HTS fabrication is costly and requires cryogenic operation, limiting practicality. In [14], a compact SIW-based dual-band filter is proposed, suitable for high-frequency integration but with limited fractional bandwidth. In [15], an interdigital filter achieves high selectivity in the C-band, yet its design lacks independent passband tunability. In [16], a dual-band filter with reflectionless characteristics and enhanced stopband suppression is presented. While their design excels in out-of-band reflection absorption, it employs a more complex topology with additional matching networks. In contrast, our proposed resonator provides independent control of both passbands through adjustable stub parameters, achieves low loss on standard PCB, and maintains a compact footprint suitable for 5G front-end modules.

In this paper, a multimode resonator is designed, which consists of a uniform half-wavelength transmission line loaded with two open-circuited stubs of different impedances at symmetric central positions. The proposed filter possesses excellent out-of-band rejection capability and demonstrates performance advantages compared to other similar filters. This paper analyzes the resonance characteristics of the multimode resonator and verifies the feasibility of the parameter mechanism of “overall tuning by the main stub + targeted regulation by the auxiliary stubs”.

## 2. Design and Analysis of the Resonator

### 2.1. Structure of the Multimode Resonator

**Figure 1:**
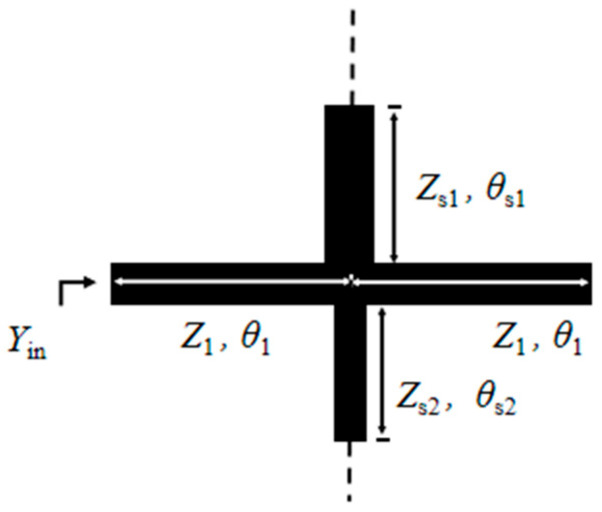
Structure diagram of the multimode resonator.

The structure of the novel multimode resonator is shown in Figure 1. It consists of a uniform half-wavelength transmission line loaded with two open-circuited short stubs at upper and lower symmetric central positions. The impedance and electrical length of the uniform half-wavelength transmission line are defined as *Z*_1_ and *θ*_1_, respectively. The impedance and electrical length of the upper stub are *Z*_s1_ and *θ*_s1_, while those of the lower stub are *Z*_s2_ and *θ*_s2_.

### 2.2. Resonance Analysis of the Resonator

**Figure 2:**
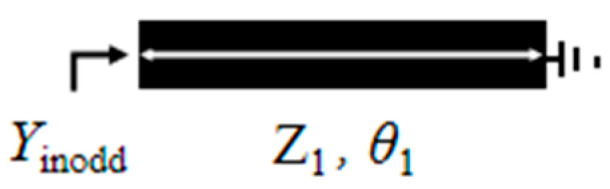
Odd-mode structure diagram.

**Figure 3:**
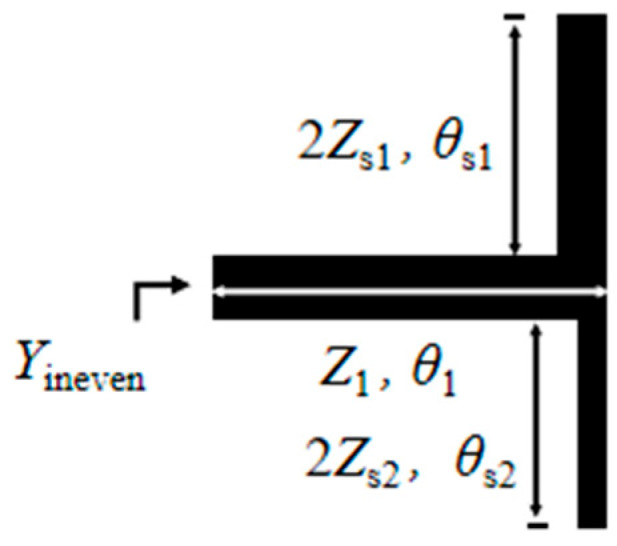
Even-mode structure diagram.

Define the impedance ratio *K*_1_ as *K*_1_ = *Z*_s1_/*Z*_1_ and *K*_2_ as *K*_2_ = *Z*_s2_/*Z*_1_. Based on transmission line theory, the input admittance Y_in_ of the resonator can be derived. The solutions satisfying the condition Y_in_ = 0 correspond to the resonant modes of the resonator. Figure 2 presents the odd-mode structure diagram of the novel multimode resonator, and Figure 3 shows the even-mode structure diagram. Based on the odd-even mode analysis method in transmission line theory [16], the resonance conditions corresponding to the odd-mode and even-mode resonances of the novel multimode resonator can be expressed as follows:

(1) $$-j\tan\theta_{1}=0$$

(2) $$K_{2}\tan\theta_{s1}+K_{1}\tan\theta_{s2}+2K_{1}K_{2}\tan\theta_{1}=0$$

The resonator has three electrical lengths: *θ*_1_, *θ*_s1_, and *θ*_s2_. Define the electrical length ratios α_1_ and α_2_. As α_1_ = 2*θ*_s1_/*θ_T_* and α_2_ = 2*θ*_s2_/*θ*_T_, where *θ*_T_ is the total electrical length of the uniform half-wavelength transmission line (*θ*_T_ = 2*θ*_1_). Substituting the two electrical length ratios into Equations (1) and (2), the resonance conditions corresponding to the odd-mode and even-mode resonances are obtained as follows:

(3) $$-jctg\left(\frac{1}{2}\theta_{T}\right)=0,$$

(4) $$K_{2}\tan\left(\frac{\alpha_{1}}{2}\theta_{T}\right)+K_{1}\tan\left(\frac{\alpha_{2}}{2}\theta_{T}\right)+2K_{1}K_{2}\tan\left(\frac{1}{2}\theta_{T}\right)=0.$$

It can be observed from Equation (3) that the odd-mode resonant frequency of the proposed resonator is independent of the loaded stubs. From Equation (4), the even-mode resonant frequency is related to the electrical lengths *θ*_s1_ and *θ*_s2_ of the loaded stubs. Therefore, this characteristic can be utilized to design a frequency-controllable dual-band microstrip bandpass filter. The design concept is: first, adjust the odd-mode resonant frequency to the desired operating frequency, and then tune the even-mode resonant frequency to the target value by modifying the structural parameters of the two stubs. Compared with the traditional SIR, the proposed resonator has more structural parameters affecting the resonance conditions, leading to a significant improvement in design flexibility and the ability to independently adjust the resonant frequencies of the two passbands.

## 3. Filter Design and Parameter Optimization

### 3.1. Filter Structure

**Figure 4:**
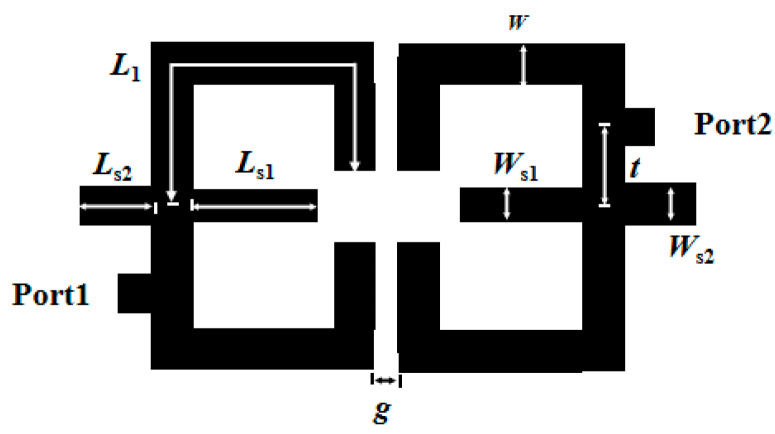
Structure diagram of the dual-band filter.

To further verify the above analysis, a dual-band microstrip bandpass filter is designed using the aforementioned multimode resonator. Electrical coupling is adopted as the coupling method for the filter, and a tapped line feeding method is employed. Out-of-band rejection is a crucial performance indicator of filters, so transmission zeros are introduced to enhance out-of-band rejection. Through the selected electrical coupling structure and tapped 0° feeding method, combined with simulation analysis and parameter optimization using IE3D15 software, the structure diagram of the dual-band microstrip bandpass filter based on the multimode resonator is finally obtained, as shown in Figure 4. Both simulation and measurement of the proposed filter were conducted on an RT/Duroid 5880 substrate, which is characterized by a thickness of 0.787 mm, a relative dielectric constant (ε_r_) of 2.2, and a loss tangent of 0.0009.

**Figure 5:**
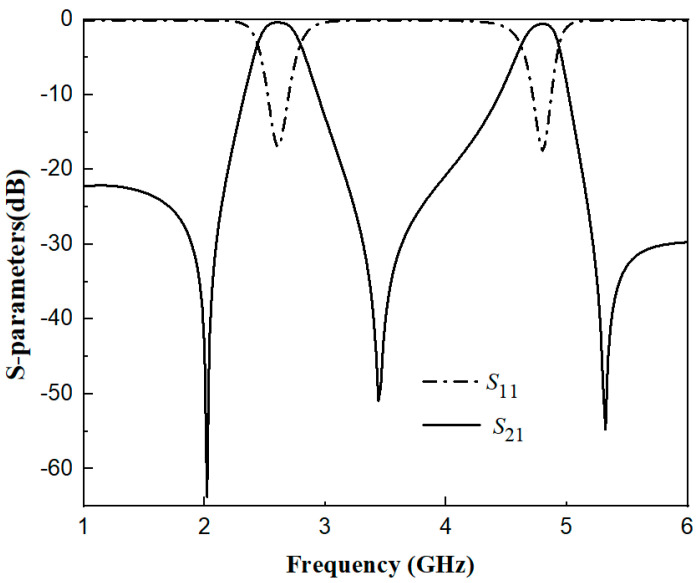
Performance diagram of the dual-band filter.

By adjusting the electrical length ratios of the upper and lower stubs, the dual-band filter is realized. Figure 5 shows the performance diagram of the dual-band filter obtained through HFSS simulation, with center frequencies of 2.6 GHz and 4.8 GHz. The simulation parameters of the filter are as follows: *L*_1_ = 22.2 mm, *W* = 1.8 mm, *W*_s1_ = 0.45 mm, *L*_s1_ = 6.8 mm, *L*_s2_ = 0.8 mm, *W*_s2_ = 0.7 mm, *t* = 5.5 mm and *g* = 0.2 mm. For the first passband (center frequency: 2.6 GHz), the return loss |*S*_11_| is 16.3 dB, the insertion loss |*S*_21_| is 0.358 dB, and the 3 dB bandwidth is 13.96%. For the second passband (center frequency: 4.8 GHz), the return loss |*S*_11_| is 16.8 dB, the insertion loss |*S*_21_| is 0.558 dB, and the 3 dB bandwidth is 6.7%. Due to the combined effect of the electrical coupling structure and 0° feeding adopted in the design, three transmission zeros are generated at frequencies of 2.02 GHz, 3.44 GHz, and 5.31 GHz, achieving high selectivity.

### 3.2. Weak Coupling Analysis of Each Stub

**Figure 6:**
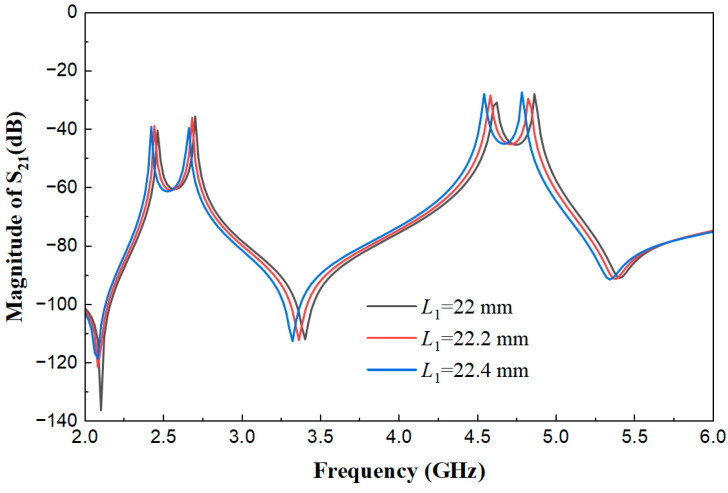
Weak coupling effect diagrams of the multimode resonator under different *L*_1_ values.

Figure 6 shows the weak coupling effect diagrams of the multimode resonator when *L*_1_ is 22 mm, 22.2 mm, and 22.4 mm, with other parameters fixed (*W*_s1_ = 0.45 mm, *L*_s1_ = 6.8 mm, *L*_s2_ = 0.8 mm, *W*_s2_ = 0.7 mm, *W* = 1.8 mm, *t* = 5.5 mm, *g* = 0.2 mm). It can be observed from Figure 6 that as the main resonant length *L*_1_ increases from 22 mm to 22.4 mm, all resonant modes of the resonator shift uniformly towards lower frequencies, while the frequency spacing between modes and the coupling strength remain relatively stable. This indicates that *L*_1_, as the main resonant length of the resonator, primarily functions to globally control the positions of the fundamental frequency and each harmonic frequency, providing a foundation for the precise tuning of the center frequencies of the dual-band filter.

**Figure 7:**
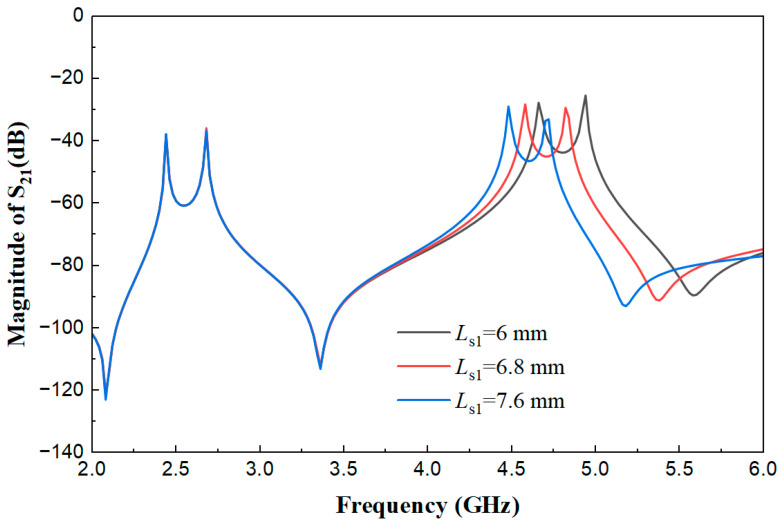
Weak coupling effect diagrams of the multimode resonator under different *L*_s1_ values.

Figure 7 presents the weak coupling effect diagrams of the multimode resonator when *L*_s1_ is 6 mm, 6.8 mm, and 7.6 mm, with other parameters fixed (*W*_s1_ = 0.45 mm, *L*_1_ = 22.2 mm, *L*_s2_ = 0.8 mm, *W*_s2_ = 0.7 mm, *W* = 1.8 mm, *t* = 5.5 mm, *g* = 0.2 mm). As shown in the figure, when Ls1 is 6 mm, 6.8 mm, and 7.6 mm, the characteristics of the low-frequency resonant peak near 2.5 GHz do not change significantly, but the overlap degree and amplitude distribution of the high-order resonant peaks near 5 GHz change obviously. This indicates that *L*_s1_ is a key parameter for targeted regulation of the multimode coupling matching state in the high-frequency band.

**Figure 8:**
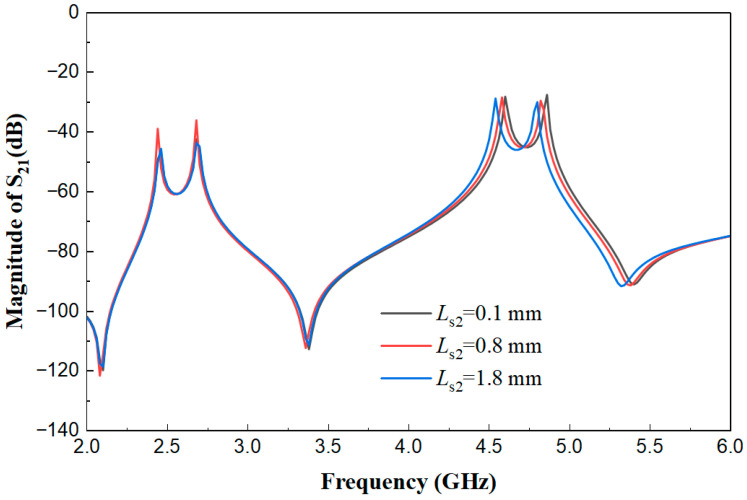
Weak coupling effect diagrams of the multimode resonator under different *L*_s2_ values.

**Figure 9:**
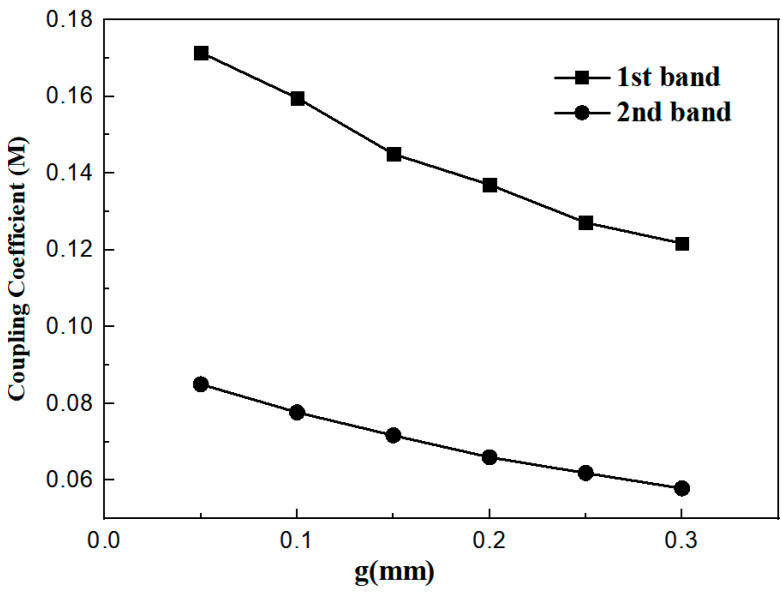
Coupling coefficient diagram of the two passbands.

Figure 8 displays the weak coupling effect diagrams of the multimode resonator when Ls2 is 0.1 mm, 0.8 mm, and 1.8 mm, with other parameters fixed (*L*_1_ = 22.2 mm, *W* = 1.8 mm, *W*_s1_ = 0.45 mm, *L*_s1_ = 6.8 mm, *W*_s2_ = 0.7 mm, *t* = 5.5 mm, *g* = 0.2 mm). It can be seen from Figure 9 that the variation in the coupling segment *L*_s2_ mainly affects the high-frequency resonant mode (near 5.0 GHz), and its function is similar to that of *L*_s1_ but acts on different high-frequency resonant points. This indicates that *L*_s1_ and *L*_s2_ are jointly responsible for the independent regulation of different resonant modes within the high-frequency band.

This parameter mechanism of “overall tuning by the main stub + targeted regulation by the auxiliary stubs” endows the resonator with flexible regulation capability of multimode characteristics, providing a reliable basic unit for the multi-frequency characteristic design of dual-band filters.

### 3.3. Coupling Coefficient

In this paper, two novel multimode resonators are electrically coupled to realize a dual-band bandpass filter with center frequencies of 2.6 GHz and 4.8 GHz, respectively. The 3 dB fractional bandwidths (FBW) of the two passbands are 13.96% and 6.7%, respectively. The lumped circuit element values of the corresponding low-pass prototype filter are: *g*_1_ = 0.90467 and *g*_2_ = 1.25868, where *g*_n_ (n = 1 to 2) are the element values [16]. The theoretical coupling coefficient and external quality factor can be obtained by the following formulas [17]:

(5) $$M_{i,j}=FBW/\sqrt{g_{1}g_{2}}$$

(6) $$Q_{e}=g_{1}g_{2}/FBW$$

Substituting the bandwidths, g_1_, and g_2_ values of the two passbands into Equations (5) and (6), the following results are obtained:

$$M_{ij}^{I}=\left[\begin{array}{cc} 0 & 0.13\\ 0.13 & 0 \end{array}\right]\;\;\;\;\;\;\mathrm{at}\;2.6\;\mathrm{GHz}$$

$$M_{ij}^{II}=\left[\begin{array}{cc} 0 & 0.05\\ 0.05 & 0 \end{array}\right]\;\;\;\;\;\;\mathrm{at}\;4.8\;\mathrm{GHz}$$

The external quality factor of the first passband is $Q_{e}^{I}=8.16$, and that of the second passband is $Q_{e}^{II}=0.08$.

**Figure 10:**
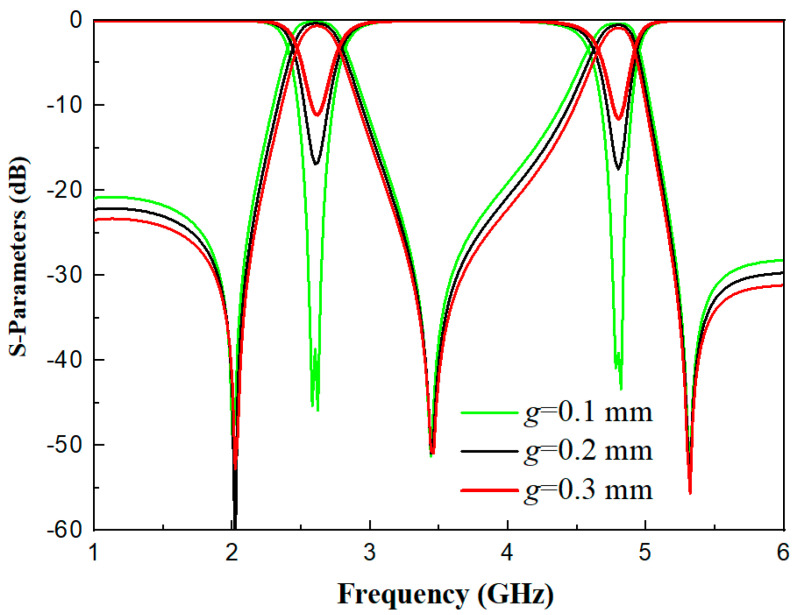
Comparison diagram of S-parameters under different *g* values.

Studies have shown that *t* influences the external quality factor, and the value of the coupling coefficient related to the desired fractional bandwidth is determined by the coupling gap (*g*). In addition, the coupling coefficient can be calculated through the transmission coefficient obtained from full-wave simulation [17], and its expression is as follows:

(7) $$K_{i,j}=\frac{{f_{H}}^{2}-{f_{L}}^{2}}{{f_{H}}^{2}+{f_{L}}^{2}}.$$

where *f*_H_ and *f*_L_ are defined as the frequencies corresponding to the higher-order mode and lower-order mode among the two resonant modes, respectively, and *f*_1_ is the resonant frequency. As can be seen from Figure 9, as the value of *g* increases, the value of the coupling coefficient decreases. In this paper, *g* is set to 0.2 mm because the minimum precision of the laboratory engraving machine is 0.2 mm. Figure 10 shows the comparison diagram of S-parameters under different *g* values. It can be observed that as *g* decreases, the insertion loss |*S*_21_| decreases, while the return loss increases.

### 3.4. Influence of Two Open-Circuited Stubs on Filter Performance

**Figure 11:**
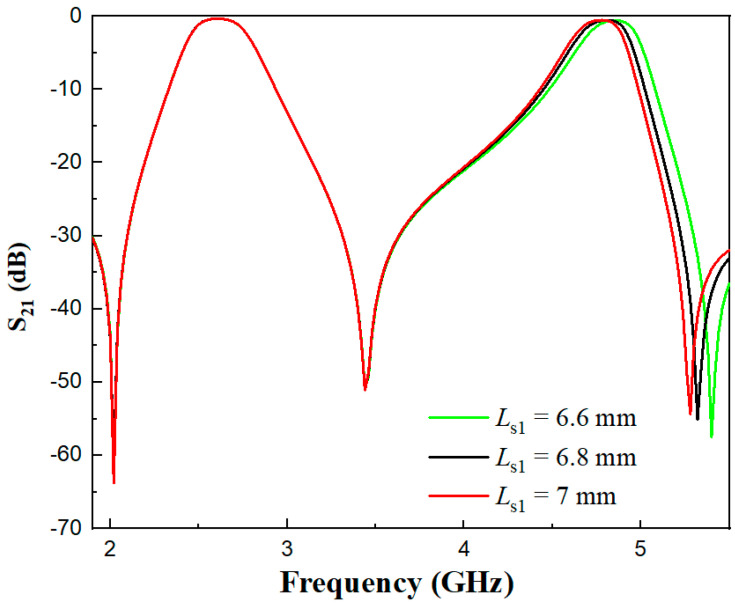
Comparison diagram of S_21_ under different *L*_s1_ values.

**Figure 12:**
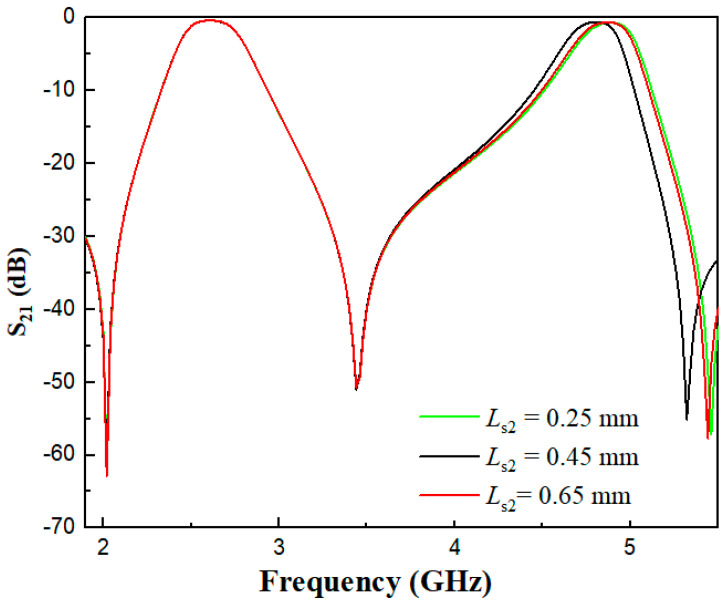
Comparison diagram of S_21_ under different *L*_s2_ values.

As can be seen from Figure 11 and Figure 12, the two open-circuited stubs *L*_s1_ and *L*_s2_ have no influence on the first passband, which verifies Equation (3) that the odd-mode resonant frequency is independent of the two stubs. In the even-mode analysis, Equation (4) is a function of the electrical length ratios α1 and α2 and impedance ratios. With fixed impedance ratios, the even-mode resonance depends on the electrical length ratios. It can be observed from Figure 11 that as *L*_s1_ increases, the center frequency of the second passband decreases. Figure 12 shows that *L*_s2_ plays a fine-tuning role in the center frequency of the second passband.

**Figure 13:**
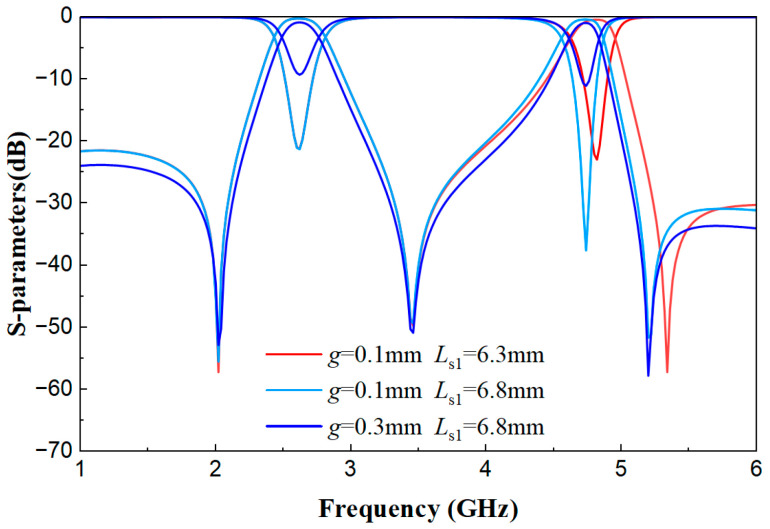
Comparison diagram of S-parameters under different *g* and *L*_s1_ values.

Figure 13 is shown below. When *g* = 0.1 mm (strong coupling), increasing *L*_s1_ from 6.3 mm to 6.8 mm improves the *S*_21_ performance of the second passband. When switching from strong coupling (*g* = 0.1 mm) to weak coupling (*g* = 0.3 mm) while keeping *L*_s1_ unchanged, the *S*_11_ and *S*_21_ performance of both passbands deteriorates.

By summarizing Section 3.1, Section 3.2, Section 3.3 and Section 3.4 content, we can derive the sensitivity (analysis results):

(1) *L*_1_ (Main Transmission Line Length): It has the highest sensitivity to the center frequencies of the dual passbands and serves as a global core parameter. It must be fixed first to ensure a stable frequency baseline.
(2) *g* (Coupling Gap): It has the second-highest sensitivity to coupling strength and insertion loss, directly determining the passband matching foundation. It should be optimized after *L*_1_ is fixed.
(3) *L*_s1_ (Upper Open-Circuited Stub Length): It has extremely high sensitivity to the insertion loss optimization of the high-frequency passband and acts as a core fine-tuning parameter for the performance of the high-frequency passband.
(4) *L*_s2_ (Lower Open-Circuited Stub Length): It has the lowest sensitivity and is only used for fine calibration of the high-frequency passband, with a limited impact on overall performance.

### 3.5. Procedure for Calculating the Filter’s Parameters

To facilitate practical application of the proposed filter, this section presents a generalizable step-by-step procedure for calculating parameters tailored to arbitrary dual-band center frequencies (*f*_1_, *f*_2_) and target performance indicators.

Step 1: Define Design Requirements and Substrate Parameters

Define core performance metrics: Target first passband center frequency *f*_1_, second passband center frequency *f*_2_, 3 dB fractional bandwidths (FBW1, FBW2), maximum allowable insertion loss (IL), and minimum required return loss (RL). Specify substrate parameters: Relative dielectric constant, substrate thickness, and loss tangent. For consistency with the proposed filter, the RT/Duroid 5880 substrate is referenced here (a thickness of 0.787 mm, a relative dielectric constant (ε_r_) of 2.2, and a loss tangent of 0.0009), but the procedure is adaptable to other substrates.

Step 2: Tune Odd-Mode Resonance to Target First Passband (*f*_1_)

The resonator’s odd-mode resonant frequency is independent of the loaded stubs (derived from Equation (3)), so *f*_1_ = *f*_odd_. Calculate the total electrical length of the uniform half-wavelength transmission line: $\theta_{T}=90^{\circ }$ (at *f*_1_, where $\theta_{T}=2\theta_{1}$ and $\theta_{1}$ denotes the electrical length of the transmission line segment). Convert $\theta_{T}$ to the physical length *L*_1_ of the main transmission line: *L*_1_$\;=\lambda_{0}/2$, where $\lambda_{0}$ is the effective wavelength on the substrate.

Step 3: Tune Even-Mode Resonance to Target Second Passband ($f_{2}$)

The even-mode resonant frequency is governed by the two symmetric open-circuited stubs (Equation (4)). Define electrical length ratios $\alpha_{1}=2\theta_{s1}/\theta_{T}$ and $\alpha_{2}=2\theta_{s2}/\theta_{T}$, where $\theta_{s1}$ and $\theta_{s2}$ are the electrical lengths of the upper ($Z_{s1}$) and lower ($Z_{s2}$) stubs, respectively.

Step 4: Calculate Coupling Coefficient and Determine Coupling Gap g

Employ a low-pass prototype filter with element values $g_{1}=0.90467$ and $g_{2}=1.25868$ (consistent with the proposed design). Compute the required coupling coefficient k for each passband using the fractional bandwidth.

The coupling gap g between the two multimode resonators is determined by the target *K* (Figure 9). For the RT/Duroid 5880 substrate, g=0.2 mm (constrained by fabrication tolerance) achieves K≈0.13 (for $f_{1}=2.6\;GHz$) and K≈0.07 (for $f_{2}=4.8\;GHz$), matching typical FBW requirements.

Step 5: Optimize Stub Impedances ($Z_{s1}$, $Z_{s2}$) and Widths ($W_{s1}$, $W_{s2}$)

Set the main transmission line impedance $Z_{1}=50\;\Omega$. Select $Z_{s1}$ and $Z_{s2}$ to satisfy the impedance ratios $K_{1}$ and $K_{2}$. For example, $Z_{s1}=119\;\Omega$ corresponds to a width $W_{s1}=0.45\;mm$, and $Z_{s2}=99\;\Omega$ corresponds to $W_{s2}=0.7\;mm$ on the RT/Duroid 5880 substrate.

Step 6: Full-Wave Simulation Verification and Fine-Tuning

Import the initial set of parameters (*L*_1_, *L*_s1_, *L*_s2_, *W*, *W*_s1_, *W*_s2_, *g*) into electromagnetic simulation software (e.g., IE3D15). Fine-tune these parameters to minimize IL, maximize RL, and ensure three transmission zeros (TZs) are generated between and adjacent to the passbands (e.g., 2.02 GHz, 3.44 GHz, and 5.31 GHz for the proposed filter) to achieve high selectivity.

This procedure facilitates the design of dual-band filters with arbitrary center frequencies while retaining the core merits of low IL, compact footprint, and independent passband tunability.

## 4. Experimental Results

**Figure 14:**
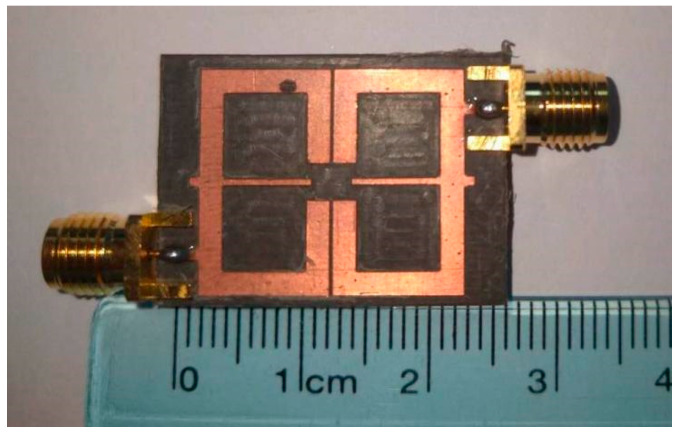
Photograph of the physically fabricated dual-band filter.

The filter prototype was fabricated on an RT/Duroid 5880 substrate (thickness 0.787 mm, relative permittivity ε_r_ = 2.2, loss tangent tanδ = 0.0009), with the layout designed via IE3D15 and exported as Gerber files for ultraviolet lithography—including substrate cleaning, photoresist coating, exposure, development, etching, and photoresist removal—resulting in a final sample size of 20.8 mm × 18.1 mm, with 50 Ω microstrip probes soldered to input/output ports for impedance matching. The physically manufactured dual-band filter is illustrated in Figure 14, with an overall footprint of 20.8 mm × 18.1 mm—corresponding to approximately 0.185 λ_0_ × 0.161 λ_0_, where λ_0_ represents the free-space wavelength at the first passband’s center frequency. Following successive iterations of simulation optimization, the structural parameters of the dual-band filter were ultimately finalized as *L*_1_ = 22.2 mm, *W* = 1.8 mm, *W*_s1_ = 0.45 mm, *L*_s1_ = 6.8 mm, *L*_s2_ = 0.8 mm, *W*_s2_ = 0.7 mm, *t* = 5.5 mm, and *g* = 0.2 mm. For testing, an Agilent N5247A vector network analyzer (VNA, 10 MHz–50 GHz) paired with a SOLT calibration suite and microwave anechoic chamber was used. For the tested filter prototype, the first passband features a center frequency of 2.6 GHz, a return loss (|*S*_11_|) of 13.7 dB, an insertion loss (|*S*_21_|) of 0.37 dB, and a 3 dB bandwidth of 17.3%. Meanwhile, the second passband exhibits a center frequency of 4.8 GHz, a return loss (|*S*_11_|) of 23.6 dB, an insertion loss (|*S*_21_|) of 0.77 dB, and a 3 dB bandwidth of 8.75%. Owing to the synergistic effect of electrical coupling and the 0° feeding scheme integrated into the filter structure, three transmission zeros are generated at 2 GHz, 3.45 GHz, and 5.45 GHz, thereby enabling the device to attain high selectivity.

**Figure 15:**
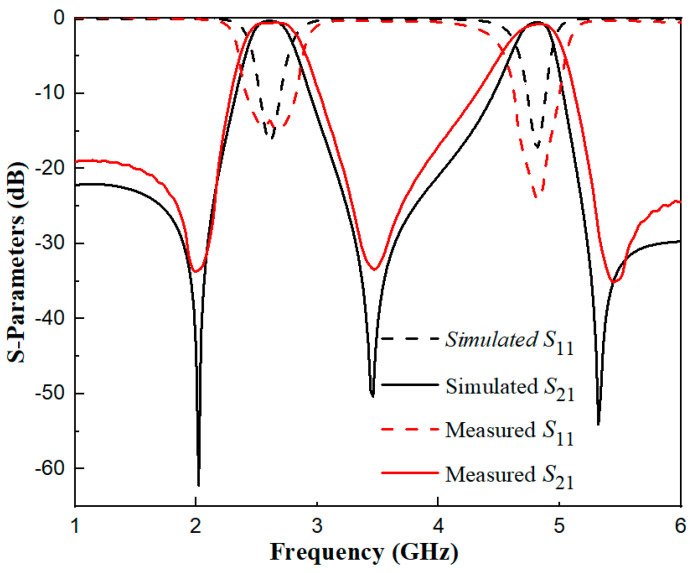
Comparison diagram between simulation and measurement results of the dual-band filter.

Figure 15 depicts a comparative plot of the dual-band filter’s simulation and measured results. As illustrated in this figure, the simulated outcomes exhibit good agreement with the experimental measurement data.

Table 1 presents a performance comparison between the dual-band filter proposed in this paper and those reported in other reference literature. While each design operates at different center frequencies—making direct comparison of absolute values challenging—the inclusion of the normalized frequency ratio (*f*_2_/*f*_1_) and circuit size in guided wavelengths (λ_0_ × λ_0_) enables a fair assessment of design efficiency and compactness. Among the compared designs, the proposed filter achieves remarkably low insertion losses of 0.37 dB and 0.77 dB in its two passbands, which is superior to several references such as [9] (2.68/2.93 dB) and [12] (1.05/1.86 dB). Additionally, with a normalized circuit size of only 0.185λ_0_ × 0.161λ_0_, it demonstrates exceptional compactness compared to larger layouts like [10] (0.75 × 0.4 λ_0_) and [11] (0.31 × 0.46 λ_0_). Although some designs, such as [12], also exhibit small footprints, the proposed filter maintains better insertion loss and a useful frequency ratio (1.84), comparable to [2,3]. Thus, the proposed work stands out for its optimal balance of low loss, miniaturized layout, and flexible frequency separation, making it a competitive candidate for modern multi-band communication systems.

## 5. Conclusions

This paper presents a novel dual-band bandpass filter based on a multimode resonator loaded with two open-circuited stubs of different impedances. The resonator allows independent control of the two passbands through the “main stub + auxiliary stubs” tuning mechanism. The filter exhibits low insertion loss (0.37 dB and 0.77 dB), good return loss, and high selectivity with three transmission zeros. Measured results agree well with simulations. The design is compact (0.185λ_0_ × 0.161λ_0_), suitable for 5G communication systems, and can be extended to other frequency pairs through the provided design procedure. Future work may include tunable or reconfigurable versions of the proposed structure.

## Figures and Tables

**Table 1:** Performance Comparison of Different Dual-Band Filters.

	1st/2nd Passband Center Frequency/GHz	Normalized Frequency Ratio (*f*_2_/*f*_1_)	|*S*_21_|/dB	|*S*_11_|/dB	FBW/%	Circuit Size/(λ_0_ × λ_0_)
[2]	7.95/14.65	1.84	<0.7		64.5/29.4	2.55 × 1.1
[3]	1.45/2.67	1.84	0.92/0.96	>14		0.34 × 0.25
[9]	1.55/3.335	1.09	2.68/2.93	>9.23	19.4/2.7	0.22 × 0.25
[10]	0.37/1.54	4.16	<0.4	>20	0.2/0.27	0.75 × 0.4
[11]	1.25/1.9	1.52	0.77/0.5		7.6/8.4	0.31 × 0.46
[12]	2.34/3.72	1.59	1.05/1.86	>20	15.8/8.33	0.14 × 0.16
This Work	2.6/4.8	1.84	0.37/0.77	13.7/23.6	13.65/6.4	0.185 × 0.161

## Data Availability

The data presented in this study are available on request from the corresponding author.
